# Transcriptional Profiling of Immune-Related Genes in *Leishmania infantum*-Infected Mice: Identification of Potential Biomarkers of Infection and Progression of Disease

**DOI:** 10.3389/fcimb.2018.00197

**Published:** 2018-06-26

**Authors:** Eduardo Ontoria, Yasmina E. Hernández-Santana, Ana C. González-García, Manuel C. López, Basilio Valladares, Emma Carmelo

**Affiliations:** ^1^Instituto Universitario de Enfermedades Tropicales y Salud Pública de Canarias, Universidad de La Laguna, La Laguna, Spain; ^2^Departamento de Biología Molecular, Instituto de Parasitología y Biomedicina “López Neyra”, Consejo Superior de Investigaciones Científicas, Granada, Spain

**Keywords:** *Leishmania infantum*, transcriptional profiling, high-throughput qPCR, immune responses, regression models, biomarkers

## Abstract

*Leishmania* spp. is a protozoan parasite that affects millions of people around the world. At present, there is no effective vaccine to prevent leishmaniases in humans. A major limitation in vaccine development is the lack of precise understanding of the particular immunological mechanisms that allow parasite survival in the host. The parasite-host cell interaction induces dramatic changes in transcriptome patterns in both organisms, therefore, a detailed analysis of gene expression in infected tissues will contribute to the evaluation of drug and vaccine candidates, the identification of potential biomarkers, and the understanding of the immunological pathways that lead to protection or progression of disease. In this large-scale analysis, differential expression of 112 immune-related genes has been analyzed using high-throughput qPCR in spleens of infected and naïve Balb/c mice at four different time points. This analysis revealed that early response against *Leishmania* infection is characterized by the upregulation of Th1 markers and M1-macrophage activation molecules such as *Ifng, Stat1, Cxcl9, Cxcl10, Ccr5, Cxcr3, Xcl1*, and *Ccl3*. This activation doesn't protect spleen from infection, since parasitic burden rises along time. This marked difference in gene expression between infected and control mice disappears during intermediate stages of infection, probably related to the strong anti-inflammatory and immunosuppresory signals that are activated early upon infection (*Ctla4*) or remain activated throughout the experiment (*Il18bp*). The overexpression of these Th1/M1 markers is restored later in the chronic phase (8 wpi), suggesting the generation of a classical “protective response” against leishmaniasis. Nonetheless, the parasitic burden rockets at this timepoint. This apparent contradiction can be explained by the generation of a regulatory immune response characterized by overexpression of *Ifng, Tnfa, Il10*, and downregulation *Il4* that counteracts the Th1/M1 response. This large pool of data was also used to identify potential biomarkers of infection and parasitic burden in spleen, on the bases of two different regression models. Given the results, gene expression signature analysis appears as a useful tool to identify mechanisms involved in disease outcome and to establish a rational approach for the identification of potential biomarkers useful for monitoring disease progression, new therapies or vaccine development.

## Introduction

The term leishmaniasis includes a spectrum of diseases caused by parasites belonging to genus *Leishmania*, with symptoms ranging from cutaneous lesions to fatal visceral leishmaniosis (VL) the most severe clinical form of the disease. The organs commonly affected during VL are the bone marrow, liver, and spleen and clinical symptoms include hepatosplenomegaly, long-term, low-grade fever, muscle wasting, anemia, leukopenia, polyclonal hypergammaglobulinemia, and weight loss (reviewed in Alvar et al., 1997).

*Leishmania* parasites are obligate intracellular pathogens in the mammalian host and therefore a successful T cell-dependent immune response is required to control infection. During many years, disease outcome was thought to be driven by the Th1/Th2 paradigm of resistance/susceptibility (Heinzel et al., 1989). However, identification of new cell populations, including CD4^+^ T cell regulatory (Treg) populations, as well as further CD4^+^ T helper (Th) populations like Th17, Th9, and T follicular helper (Tfh) cells, have certainly questioned the simplicity of the Th1/Th2 paradigm to intracellular infection (Bettelli et al., 2006; Korn et al., 2009; Jäger and Kuchroo, 2010; Crotty, 2011; Peterson, 2012; reviewed in Alexander and Brombacher, 2012). Successful immunity against *Leishmania* involves a complex response of several mechanisms and factors, including the migration of appropriate cell populations to the infected sites, generation of an appropriate type of immune response, cytokine microenvironment, chemokines, and others. Chemokines and their receptors have been shown to play a crucial role in determining the outcome of leishmaniasis; indeed, pathogenesis in VL is often associated with altered chemokine expression profiles and defective migration of immune cells (Stanley and Engwerda, 2006; Oghumu et al., 2010; Kong et al., 2017).

Early after infection in mice experimental model, most of the parasites appear to be phagocytized by splenic macrophages and mature DC start producing IL-12 or IL-23 to initiate protective Th1 or Th17 responses, respectively, which, in turn, will produce IFNγ, TNF or IL-17 that maximize the capacity of infected macrophages to produce NO and ROS (reviewed in Rodrigues et al., 2016). Naïve CD8 T cells are activated by DCs in the presence of IL-12 and type I IFNs and differentiate into effector cells that further contribute to the protective response by producing IFNγ and TNF (reviewed in Rodrigues et al., 2016). Nevertheless, the parasite abrogates the ability of infected DCs to initiate protective responses using several mechanisms that impair host cell function (reviewed in Arango Duque and Descoteaux, 2015; Martínez-López et al., 2018). Some of these mechanisms include exhaustion of specific CD8 T cells (in which CTLA-4 and PD-1 play a role) (reviewed in Wherry and Kurachi, 2015) and differentiation of IFN-γ and IL-10-producing Tr1 cells. In addition, spleen suffers dramatic changes in microarchitecture, including disorganization of the white and red pulp and disruption of the marginal zone, resulting in severe immunosuppression and enhancing parasite proliferation (Kaye et al., 2004; reviewed in Rodrigues et al., 2016).

One of the major hurdles for developing vaccines to either prevent or treat VL has been a limited understanding of the precise immune mechanisms required for controlling parasite growth without causing disease. Because of the intrusive techniques required to analyze tissue in VL patients, our current understanding of the host immune response during VL largely derives from studies performed in genetically susceptible mice or hamsters infected with viscerotropic species (Faleiro et al., 2014; Kong et al., 2017; Medina-Colorado et al., 2017). These animals develop distinct, organ specific immune responses as disease progresses (Engwerda and Kaye, 2000; Rodrigues et al., 2016). In the spleen, chronic infection leads to splenomegaly and results in structural alterations in the architecture of the spleen tissue which are thought to contribute to immune suppression in this organ during VL (reviewed in Faleiro et al., 2014; Rodrigues et al., 2016).

*Leishmania* has developed several mechanisms that influence macrophages leishmanicidal activity, altering the expression of genes coding for cytokines, chemokines, transcription factors, membrane receptors and molecules involved in signal transduction in infected cells. Different high-throughput techniques, such as transcriptome analysis or Serial Analysis of Gene Expression (SAGE) technology (Guerfali et al., 2008) and most recently transcriptional profiling using RNA-seq (Fernandes et al., 2016; Kong et al., 2017), have been applied to studying host-parasite interactions, providing important insights into the mechanism of pathogenesis. The use of this approach has been possible due to the good correlation between cytokine/chemokine mRNAs levels and protein expression observed in these experimental models (Kumar et al., 2010, 2014; Cuervo-Escobar et al., 2014; Zhang et al., 2017). As far as we know, our study is the first performed using high-throughput qPCR which analyses more than one hundred immune-related genes at the same time in a *L. infantum*–infection murine model, providing a large collection of differential gene expression data between infected and non-infected animals. This approach could be a useful tool to identify the mechanisms involved in disease outcome and also to establish a rational strategy for the development of immunomodulatory therapies and vaccines.

In the last few years, there is a growing need for identification of new molecules useful for monitorization of biological processes like infection or the assessment of protective immune responses. In this context, biomarkers able to predict infection or to estimate parasitic load in infected organs and its reduction upon treatment are increasingly relevant. This high-throughput approach also provides a large collection of gene expression data than can be exploited to try to identify new molecules that can be useful as biomarkers in leishmaniasis.

The aim of this work was to map global changes in gene expression patterns in the spleen, in BALB/c murine model during *Leishmania infantum* infection, particularly characterizing how immune system responds to infection. The results draw a global picture of how spleen reacts, in terms of gene expression, to leishmania infection at different timepoints. In addition, new potential biomarkers for leishmaniasis are identified, and their usefulness discussed upon the current knowledge.

## Materials and methods

### Biological samples

All experiments involving animals were conducted in accordance to both European (2010/63/UE) and Spanish legislation (Law 53/2013), after approval by the Committee for Research Ethics and Animal Welfare (CEIBA) of the University of La Laguna (Permission code: CEIBA2015-0168).

*L. infantum* (JPC strain, MCAN/ES/98/LLM-724) was maintained *in vivo* by serial murine passages. Prior to infection, amplification of amastigote-derived promastigotes, with less than 3 passages *in vitro*, was carried out by culture in RPMI medium (Gibco BRL), supplemented with 20% inactivated fetal calf serum (SBFI), 100 ug/ml streptomycin (Sigma-Aldrich, St. Louis, USA) and 100 U/ml of penicillin (Biochrom AG, Berlin, Germany) at 26°C until reaching stationary phase. Sixty-one female wild-type BALB/c mice were obtained from the breeding facilities of the Charles River Laboratories, (France) and were maintained under specific pathogen-free conditions.

### Animal infection and parasite burden determination

Mice were randomly separated in two groups: (i) non-infected control mice (*n* = 23) and (ii) mice infected with 10^6^ stationary-phase *L. infantum* promastigotes (*n* = 24) via tail vein. At week 1, 2, 4, and 8 after infection, mice (*n* = 6 per group) were euthanized by cervical dislocation, and spleen and liver portions of each mouse were collected and used for further parasitological and immunological assays. Mice were 14–15 weeks-old when challenged with *L. infantum*. Spleen samples were immediately stored in RNAlater at −80°C (Sigma-Aldrich, St. Louis, USA) for nucleic acid preservation and further mRNA extraction. Determination of parasite burden in both liver and spleen was carried out by quantitative limiting-dilution as described by Buffet et al. (1995). Fourteen more mice of the same age and origin were included for the evaluation of the potential biomarkers. These mice were divided in two groups (7 control and 7 infected mice) and all of them were infected and euthanized following the same protocols. All efforts were made to minimize animal suffering.

### RNA isolation and quantification

Total RNA isolation from *RNA later* preserved spleens (9–11 mg) was performed by cell disruption using FastPrep^®;^ System (ProScientific, Cedex, France) and Lysing Matrix D (MP Biomedicals, Solon, USA) in TRI-Reagent (Sigma-Aldrich, St. Louis, USA). RNeasy Mini Kit (Qiagen) was subsequently used for mRNA enrichment following manufacturer's instructions. Nucleic acid purity was assessed measuring OD_260/280_ and OD_260/230_ ratios using NanoDrop ND-1000 (ThermoFisher Scientific). Only samples with OD_260/280_ ratios between 2.1 and 2.2, and OD_260/230_ ratios between 1.8 and 2.2 were included in this study. RNA integrity number (RIN) was determined using 2100 Bioanalyzer (Agilent Technologies, Santa Clara, United States). RIN was >7 for all RNA samples included in this study.

### Reverse transcription and high-throughput real-time quantitative PCR (RT-qPCR)

Reverse transcription and high-throughput RT-qPCR were performed using the High Capacity cDNA Reverse Transcription kit and QuantStudio™ 12K Flex Real-Time PCR System (Thermo Fisher Scientific)^1^ according to the manufacturer's protocols, as indicated in Hernandez-Santana et al. (2016). Custom TaqMan OpenArray Real-Time PCR Plates included 112 Gene Expression Assays organized in 48 subarrays. All primers and probes were commercially designed by Thermo Fisher Scientific. The complete list of genes is shown in Table S1 of Supplemental Material. All reactions were performed in triplicate. Real-time PCR and fluorescence detection were performed using QuantStudio™ 12K Flex Real-Time PCR System (Thermo Fisher Scientific) following manufacturer's instructions, which calculates Cq values using an algorithm that takes into account the efficiency of each individual curve, called C_rt_ method. The C_rt_ method sets a threshold for each curve individually that is based on the shape of the amplification curve, regardless of the height or variability of the curve in its early baseline fluorescence. The method first estimates a curve that models the reaction efficiency from the amplification curve. It then uses this curve to determine the relative threshold cycle (C_rt_) from the amplification curve, that eliminates the need of “conventional” Real-Time PCR for calculating the efficiency of each reaction. Therefore, Cq values produced by this platform are already corrected for the efficiency of the amplification (Hernandez-Santana et al., 2016).

### Data analysis and statistics

The arithmetic average quantitative cycle (Cq) was used for data analysis. The Cq values for each qPCR run were exported from QuantStudio™ 12K Flex Real-Time PCR System, as Excel files, and imported into qBase Plus 1.3 (Biogazelle NV Zulte, Belgium) to obtain Relative Quantity (RQ) and Normalized Relative Quantity (NRQ) values from the whole data set, following manufacturer's instructions (Vandesompele et al., 2002; Hellemans et al., 2007). Two genes showed the most stable expression (*Stat6* and *Igb2*) (geNorm stability mean M-value and mean coefficient of variation lower than 0.5 and 20% respectively) and were used for normalization.

Differentially-expressed genes between infected and non-infected mice were identified using two parameters: the fold change of gene expression (FC), and the statistical significance. FC was calculated as the ratio between biological groups (infected and control mice) at each experimental timepoint and expressed as log_2_. Statistical significance was determined by the non-parametric Mann–Whitney *U*-test, considering *p* < 0.05 as statistically significant. In order to display changes, Volcano plots were made by plotting –log_10_ *p-*value on the y-axis, and log_2_ of FC on the x-axis. Genes passing both statistical significance threshold (–log_10_ *p* > 1.3, corresponding to *p* = 0.05), and biological significance threshold (log_2_ of FC > 0.6 or < −0.6, corresponding to FC > 1.5 or < −1.5), were marked in red and blue, depending on their upregulation and downregulation, respectively. Those genes were considered biologically relevant and used for further biological interpretation.

#### Development of regression models

A logistic regression model was developed in order to assess probability of infection in mice based on NRQ values of 36 genes, all of them coding for soluble molecules that could eventually be detected in blood (Table S1). From the complete pool of 61 mice used in this model, 75% of them (47 mice), were randomly selected and used for its development and 25% of them (14 mice) were used for its evaluation. The fitting of the proposed model was evaluated using *Nagelkerke* R^2^ as well as *Hosmer* and *Lemeshow* tests. The statistical significance of each selected variable was evaluated using *Wald* test. The general function for logistic regression is:

$$\begin{array}{l} p=\frac{1}{1+e^{-\left(\beta_{0}+\beta_{1\;}x_{1}+\ldots +\beta_{k\;}x_{k}\right)}} \end{array}$$

in which *p* is the probability of infection in a given individual, β_0_, β_1_, β_2_, etc., represent the regression coefficients of each variable identified by the model, and *x*_1_*, x*_2_*, x*_3_, stand for the NRQ values of each variable in that mice. Finally, the predictive power of the model, as well its sensitivity and specificity, was first auto-evaluated with those mice used for its development (47 mice) followed by an external evaluation using the extra 25% of animals (14 mice), and compared with the actual results (infected vs. non-infected), using standard formulae:

$$\begin{array}{l} sentitivity=\frac{VP}{VP+FN}\\ specificity=\frac{VN}{VN+FP} \end{array}$$

Additionally, a linear regression model was developed in order to estimate the parasitic burden in spleen in any infected mice. In this model, NRQ values for 36 genes (previously indicated, Table S1) were included as independent variables. Again, 75% of mice (23 infected mice) were used for the development of the model, and 25% of them (8 mice) were used for its evaluation. The fitting of the proposed model was evaluated using multiple correlation coefficient *R* and determination coefficient *R*^*2*^. The individual significance of each variable was analyzed using a *t*-test. The model was verified for collinearity, tolerance, linearity, normality, homoscedasticity and independence of errors. The general function for multivariant linear regression is regression is:

$$\begin{array}{l} y_{n}=\beta_{0}+\beta_{1}x_{1n\;}+\;\beta_{2}x_{2n}+\ldots +\beta_{p}x_{pn}+e_{n} \end{array}$$

in which *y* is the parasitic burden in spleen determined by the model in a given mouse, β_0_, β_1_, β_2_, etc., represent the regression coefficients of each variable identified by the model, and *x*_1_*, x*_2_*, x*_3_, etc., stand for the NRQ values of each variable in that mouse.

Statistical analyses were performed using SPSS 20 (IBM) software and the graphic representations were performed with GraphPad Prism version 5.00 (GraphPad Software, San Diego, United States).

## Results and discussion

In the last few years, differential analysis of transcriptome has raised as an increasingly relevant tool in order to identify key aspects of complex scenarios, such as infection of target organs or cells (Dillon et al., 2015; Fernandes et al., 2016; Kong et al., 2017). In this paper, an extensive real-time quantitative PCR (qPCR) analysis identified global changes in gene expression profiles of 112 immune-related genes in infected vs. non-infected spleens at four different timepoints, revealing three distinct immunopathological scenarios: early infection phase (1–2 weeks after infection), chronic phase (8 weeks after infection) and intermediate phase (4 weeks after infection) consistent with exhaustion of the immune response.

### *Leishmania infantum* early infection induces a mixed proinflammatory and immunosuppressive response in the spleens of infected mice

**Figure 1:**
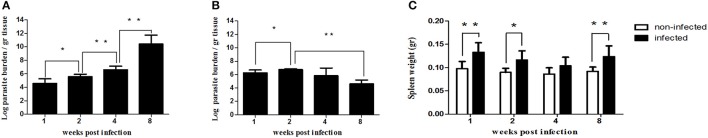
Evolution of parasite burden in spleen **(A)** and liver **(B)** of infected mice (*n* = 24). Mice were inoculated with 1 × 10^6^ promastigotes i.v. Spleen and liver parasite load were determined on week 1 (*n* = 6), week 2 (*n* = 6), week 4 (*n* = 6) and week 8 (*n* = 6) post-infection by limiting dilution assay and expressed as log_10_ of the average parasite load per gram of tissue. Evolution of spleen weight in infected (*n* = 24) vs. control mice (*n* = 23) over the course of infection **(C)**. The bars represent the weight in grams in infected (black bars) and non-infected control mice (white bars) at 1, 2, 4, and 8 weeks post-infection. Statistically significant differences are indicated (*p ≤ 0.05; **p ≤ 0.01).

One week after parasite inoculation (1 wpi), infection is clearly stablished in both spleen (10^4^ parasites/gr) and liver (10^6^ parasites/gr) (Figures 1A,B). This infection increased during the second week of infection in both organs, demonstrating that the parasite was actively replicating and overcoming the killing by the immune system (Figures 1A,B). Similarly, splenomegaly was observed in the infected groups during the first 2 weeks after infection (Figure 1C). This period corresponds to the initial phase of infection, when the immune response is apparently able to control dissemination of the parasite although not to eliminate it (Engwerda et al., 2004b; Rodrigues et al., 2016).

In order to clarify the immunological complexity of host/parasite interaction at this stage, this gene expression analysis was focused on the immunological events that occur in the spleen, being that the organ where the disease becomes chronic. A high-throughput real time quantitative PCR (qPCR) gene expression analysis was carried out in spleen samples of 47 mice, comparing RNA expression levels of 112 immune system-related genes between infected and non-infected mice. Differentially-expressed genes were identified using the fold change of gene expression (FC), and the statistical significance as described in the previous section. Only genes fulfilling both statistical and biological significance thresholds were considered biologically relevant and used for further biological interpretation.

**Figure 2:**
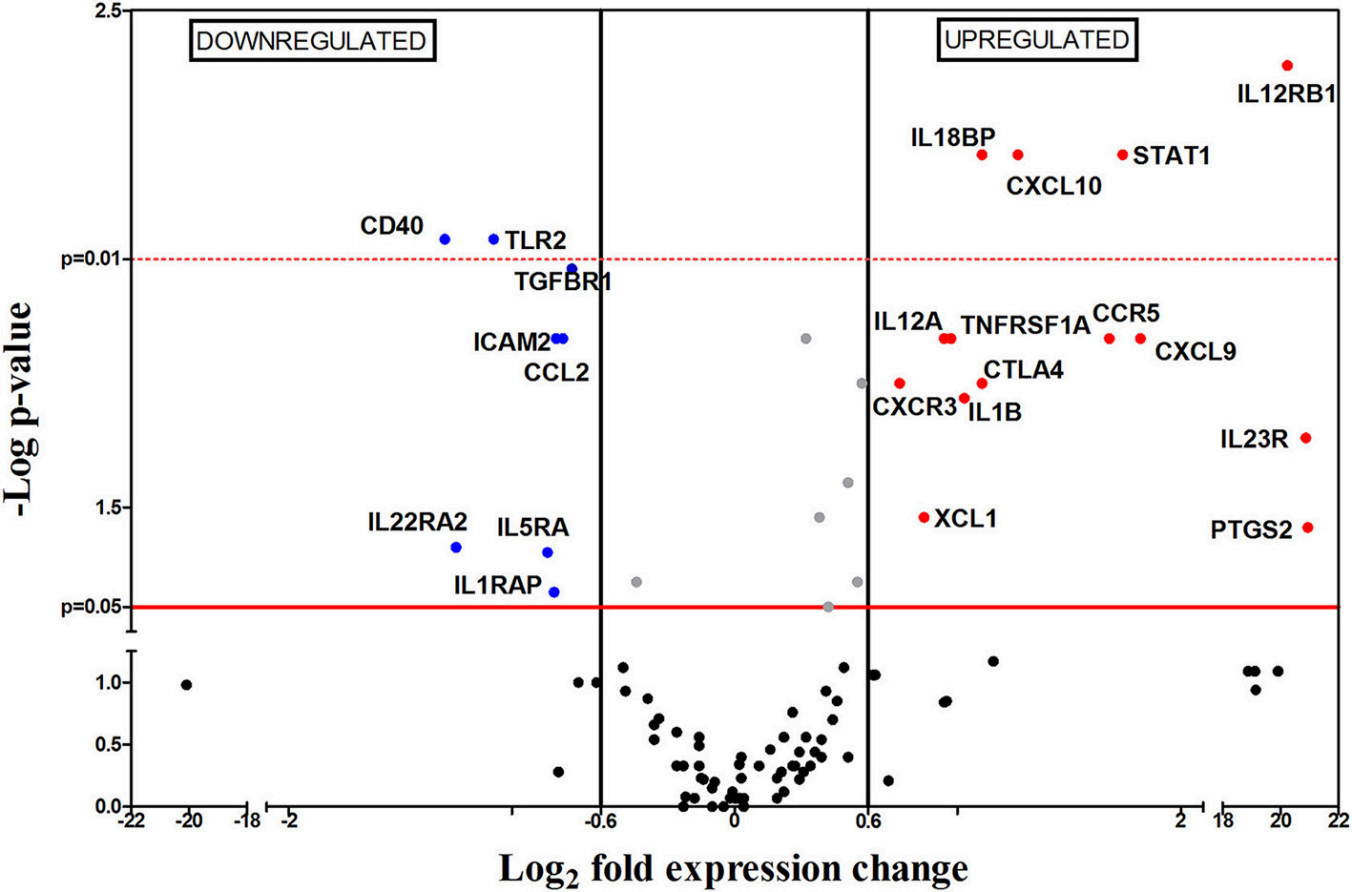
Differential gene-expression of the 112 analyzed genes in infected (*n* = 6) vs. control mice (*n* = 5), 1 wpi. The x-axis represents log_2_ of expression fold-change between infected and non-infected mice; the y-axis corresponds to the statistical significance, expressed as the negative logarithm of *p*-values. The red horizontal line indicates the cut-off for the statistical significance *p* = 0.05. Black vertical lines represent the log_2_ FC of −0.6 and 06 (corresponding to FC −1.5 and 1.5 respectively) used as biological threshold to identify differentially expressed genes. The negative values correspond to down-regulated genes (indicated in blue) and the positive values are the up-regulated genes (indicated in red). Black and gray dots represent non-differentially expressed genes.

**Figure 3:**
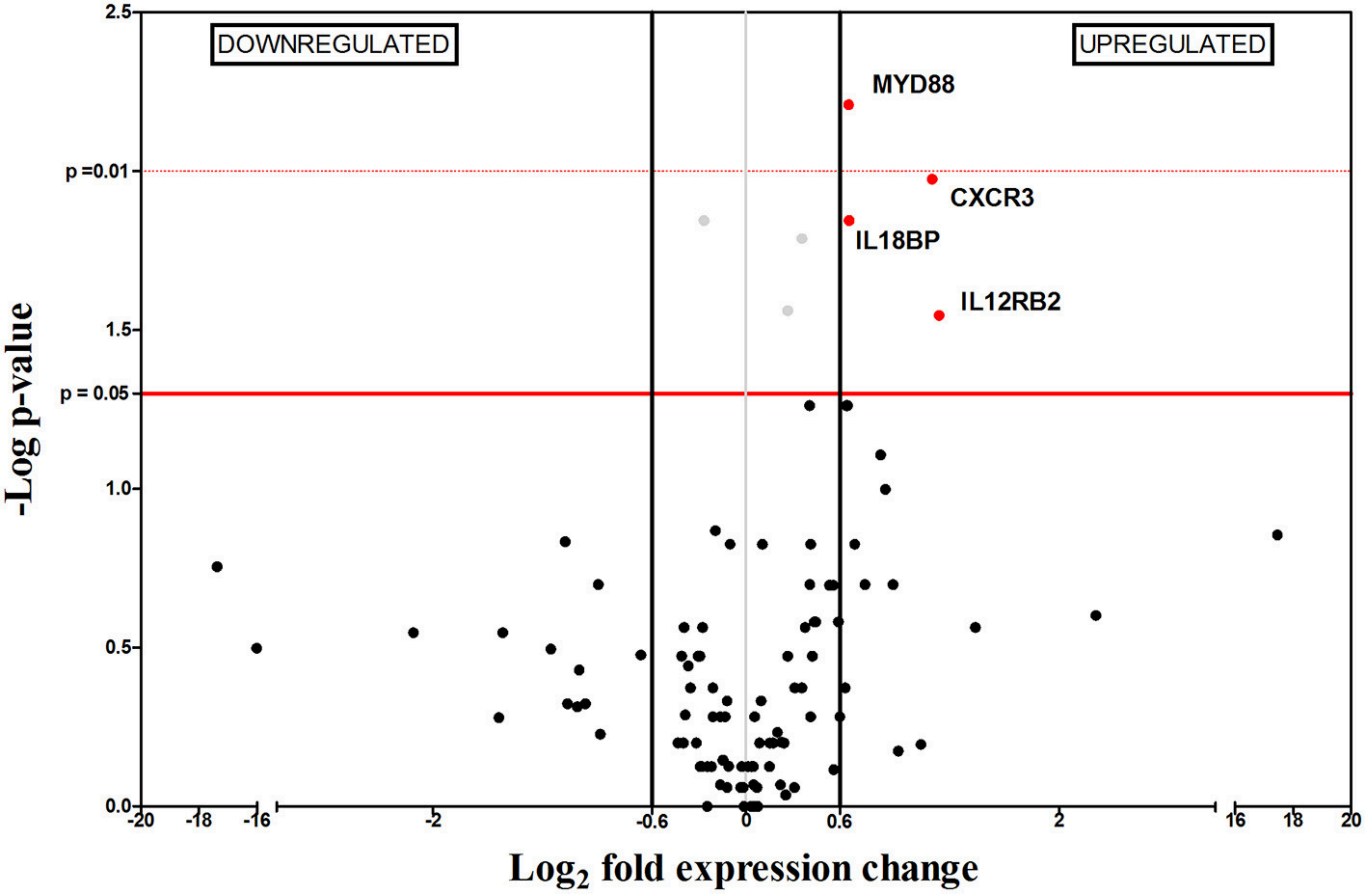
Differential gene-expression of the 112 analyzed genes in infected (*n* = 6) vs. control mice (*n* = 6), 2 wpi. The x-axis represents log_2_ of expression fold-change between infected and non-infected mice; the y-axis corresponds to the statistical significance, expressed as the negative logarithm of *p*-values. The red horizontal line indicates the cut-off for the statistical significance *p* = 0.05. Black vertical lines represent the log_2_ FC of −0.6 and 0.6 (corresponding to FC −1.5 and 1.5 respectively) used as biological threshold to identify differentially expressed genes. The negative values correspond to down-regulated genes (indicated in blue) and the positive values are the up-regulated genes (indicated in red). Black and gray dots represent non-differentially expressed genes.

One week after challenge, 22 out of the 112 genes were differentially expressed between infected and non-infected mice, most of them upregulated (71.4%, indicated as red dots in Figure 2). Within the group of upregulated mRNAs, there are genes encoding for chemokines (*Cxcl10, Cxcl9*, and *Xcl1*), chemokine receptors (*Ccr5* and *Cxcr3*), interleukins (*Il1b, Il12a, Il18bp*), interleukin receptors (*Il12rb1, Il23r*), transcription factors (*Stat1*), costimulatory-signal inhibitors (*Ctla4*) and other genes (*Tnfrsf1a* and *Ptgs2*). It is worth mentioning that three genes (*Il12rb2, Il23*r, and *Ptgs2*) are only expressed in infected mice but not in the control group, hence their high FC (Figure 3). The downregulated genes at 1 wpi were genes encoding one chemokine (*Ccl2*), four interleukin receptors (*Il22ra2, Tgfbr1, Il5ra*, and *Il1rap*), one Toll-like receptor (*Tlr2*), one costimulatory molecule (*Cd40*) and one cellular adhesion molecule (*Icam2*).

The upregulation of the chemokine receptor genes *Ccr5* and *Cxcr3*, encoding chemokine receptors CCR5 and CXCR3, which are expressed in monocytes, macrophages, immature dendritic cells (DCs), natural killer (NK) cells and activated T lymphocytes (including effector and regulatory cells) (Groom and Luster, 2011) suggested the initial recruitment of these cell populations toward spleen, in agreement with the splenomegaly observed at this timepoint (Figure 1C). Besides, CCR5 has been described as one of the *Leishmania* entry-points in macrophages, contributing to infection (Majumdar et al., 2014).

Splenomegaly is also supported by upregulation of *Xcl1* and *Cxcr3*, as well as the high transcription levels of *Cxcl9* and *Cxcl10* genes (both encoding CXCR3 ligands) observed at this timepoint, promoting the recruitment of T lymphocytes (TL) and DCs, which contribute to inflammation, as well as more CXCR3 expressing cells. In our assays, the upregulation of *Cxcr3, Cxcl9*, and *Cxcl10* suggest that the mouse immune system attempts to control infection by chemoattraction of lymphocytes and DC to the spleen. On the other hand, our data also showed downregulation on mRNA levels of *Mcp-1* or *Ccl2*, that mediates the recruitment of CC chemokine receptor 2 (CCR2) expressing cells (Ibrahim et al., 2014), which in mice includes the inflammatory monocytes subset (Gordon and Taylor, 2005; Gordon, 2007).

Taken together, these data suggest that *L. infantum* infection induces the preferential recruitment of T cells and CCR5-expressing cells toward spleen. This might be a parasite-induced strategy to escape from macrophages killing, since, according to different works the CCR5^+^ macrophage subset is prone to be silently infected by *Leishmania* through the CCR5 receptor (Bhattacharyya et al., 2008). The parasites can use this mechanism to enter silently into the macrophages and successfully establish inside the host. This would explain the existence of parasite load and the absence of expression of Th-type cytokines in our model.

Our results also revealed upregulation of two inflammation-related genes: interleukin 1 beta (*Il1b*) and prostaglandin-endoperoxide synthase (*Ptgs2*). However, we did not observe an increased expression of characteristic Th1 genes, like interleukin 12 (*Il12*) or interferon gamma (*Ifng*). The p35 gene (IL-12a subunit) is ubiquitously expressed by most cells whereas the p40 gene (IL-12b subunit) is primarily expressed by antigen presenting cells (APC) (Ma and Trinchieri, 2001) in response to different stimuli like Cd40 and Cd40-Ligand crosstalking; however *Cd40* is downregulated in our results. To be biologically active and exert its biological functions, both subunits must be present and form the heterodimer (Kima, 2008); nonetheless, as shown in Figure 2, only *Il12a* is upregulated in our experiment. These results, together with the absence of *Stat4* upregulation point to a blockade of IL-12 secretion (Yoshida et al., 2007). IL-12 acts on activated T lymphocytes, driving its differentiation to Th1 subclass, therefore its absence hampers Th1 differentiation and disease control. However IFN-γ production might also be stimulated by interleukin 18 (IL-18) (Gracie, 2003) and by NK cells after binding of lipophosphoglycan (LPG) to Toll-like receptor 2 (TLR2) on NK cells surface (Faria et al., 2012; Singh et al., 2012; Lemaire et al., 2013). In this sense, our results showed downregulation of *Tlr2*, no differential expression of *Il18* and upregulation of IL-18-binding protein coding gene (*Il18bp*) which binds IL-18 with high-affinity and inhibits its functions (Kim et al., 2000; Gracie, 2003). All these results seem to indicate another mechanism used by *Leishmania* to avoid IFN-γ production and therefore hinder generation of Th1 responses. Besides IL-12 and IFN-γ, the lack of differential expression levels of *Il4, Il13, Il5, Il17, Il23, Il21* and forkhead box P3 *(Foxp3)* genes, rule out the possibility of active Th2, Th17, nTreg and iTreg responses (Vieira et al., 2004; Gregori et al., 2012; Ma et al., 2012; Ley, 2014).

Upregulation of interleukin 12 receptor beta 1 (*Il12rb1*) and interleukin 23 receptor (*Il23r*) (whose products form the interleukin 23 (IL-23) receptor (Parham et al., 2002) might indicate and attempt to generate a Th17 response through IL-23 signaling. Nevertheless, the lack of differences on mRNA levels of transforming growth factor beta (*Tgf*β) and interleukin 6 (*Il6*), necessary for Th17 differentiation (Bettelli et al., 2006; Mangan et al., 2006; Yoshimoto et al., 2010), exclude this possibility. Taken together, the data show that, 1 wpi, a clear adaptive cellular response has not been stablished yet, and that only an inflammatory process is taking place within the spleen. However, this inflammatory profile might be counteracted by upregulation of Cytotoxic T-Lymphocyte Antigen 4 (*Ctla4)*. CTLA-4 is expressed by effector T lymphocytes upon activation as well as by Treg cells, being one of their immunosuppressive mechanisms (Gregori et al., 2012). It acts as a negative regulator of T cell activation, preventing appropriate T cell co-stimulation (Kaye et al., 1994). Therefore, its upregulation in our data suggests the existence of an immunosuppression process, as has been previously described (Murphy et al., 1998; Stanley and Engwerda, 2006). Therefore, 1 wpi the gene expression analysis reveals a mixed proinflammatory and immunosuppressive response within the spleen of the infected mice.

Two weeks after infection (2 wpi), only 4 out of the 112 genes were differentially expressed (Figure 3), probably as a consequence of the immunosuppressive signals observed at 1 wpi. This is supported by the inability to control parasite replication in target organs (Figure 1). Splenomegaly can be explained by upregulation of *Cxcr3*, indicating recruitment of a wide variety of leukocytes (reviewed in Groom and Luster, 2011).

### *L. infantum* chronic infection induces an ineffective inflammatory response in the spleens of infected mice

As previously reported (Engwerda et al., 2004a; Rodrigues et al., 2016) after 2 wpi, the parasitic burden in the liver of the infected mice starts to go down (Figure 1B) due to the development of a T-cell mediated immunity and the formation of granulomas. In contrast, the parasite population in the spleen increase slowly but steadily by 4 wpi, only to rocket by the 8th week after infection (Figure 1A). These events mark the onset of the chronic phase in visceral leishmaniasis (Engwerda et al., 2004a).

**Figure 4:**
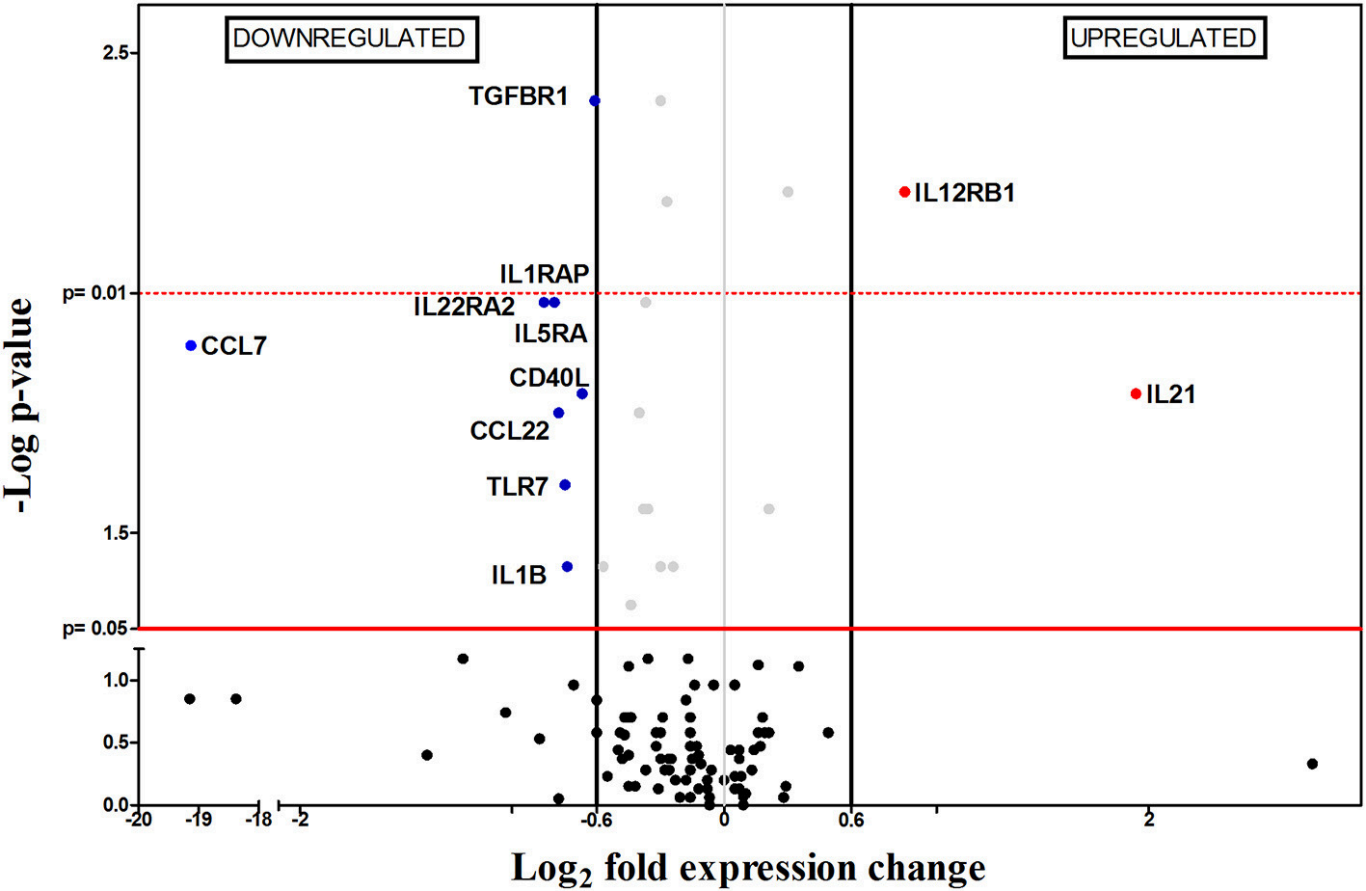
Differential gene-expression of the 112 analyzed genes in infected (*n* = 6) vs. control mice (*n* = 6), 4 wpi. The x-axis represents log_2_ of expression fold-change between infected and non-infected mice; the y-axis corresponds to the statistical significance, expressed as the negative logarithm of *p*-values. The red horizontal line indicates the cut-off for the statistical significance *p* = 0.05. Black vertical lines represent the log_2_ FC of −0.6 and 0.6 (corresponding to FC −1.5 and 1.5 respectively) used as biological threshold to identify differentially expressed genes. The negative values correspond to down-regulated genes (indicated in blue) and the positive values are the up-regulated genes (indicated in red). Black and gray dots represent non-differentially expressed genes.

In this early chronic phase (4 wpi), 11 out of the 112 genes were differentially expressed between infected and non-infected mice, only two of them (*Ccl7* and *Ccl22)* upregulated and 9/11 (82%) downregulated: four interleukin receptors (*Il22ra2, Tgfbr1, Il5ra*, and *Il1rap*), one Toll-like receptor (*Tlr7*) and one costimulatory molecule (*Cd40l*) (Figure 4). Remarkably, this was the first time along this timecourse when there was a general downregulation of gene expression of immune related genes in *Leishmania*-infected spleens. The immunosuppression process revealed 1 wpi, is displayed in our results as a reduction in the number of differentially expressed genes 2 wpi, and later as an overall downregulation of immune-related gene expression (4 wpi). This possibility is also supported by the progressive reduction of the spleen weight in the infected group between the first and the fourth week following infection (Figure 1C). This pattern is probably a consequence of impaired cell recruitment to the spleen, induced by the absence of chemokine upregulation. *L. infantum* might be blocking chemokine production in an attempt to generate an adequate environment in order to insidiously stablish infection, reflected by the slow increase (100-fold) in parasite burden during this period (Figure 1A).

Another important finding possibly related to this apparently “dormant” state of infected spleens was the strong downregulation of *Ccl7* gene expression. CCL7 is among the most pleiotropic chemokines since it recruits all major leukocyte classes, particularly monocytes and neutrophils, by binding to different chemokine receptors (CCR1, CCR2…) (Menten et al., 2001; Navas et al., 2014; Melo et al., 2017). Downregulation of *Ccl7* in infected spleen tissue, in addition to the pattern of increasing parasite burden, general downregulation of gene expression and no clear spleen inflammation, suggest a decreasing chemoattractant capacity of the immune system due to a *L. infantum*-induced immunosuppression process. This has been related to T-cell exhaustion (Joshi et al., 2009), a progressive process characterized by the loss of effector function of antigen-experienced T cells, failure to produce IFN-γ and TNF-α, and that can culminate in the physical deletion of the responding cells (Yi et al., 2010; Bhadra et al., 2011; Gigley et al., 2012; Rodrigues et al., 2014). This phenomenon can be counteracted by IL-21 produced by exhausted CD4 T cells, in an attempt to “help” the CD8 response during chronic infection (Yi et al., 2010; Gigley et al., 2012; Wherry and Kurachi, 2015). Upregulation of *Il21* is remarkable in our data (Figure 4) therefore, a scenario of *Leishmania*-induced T-cell exhaustion in spleen of the infected animals at 4 wpi seems likely, despite the lack in our data of marker genes like programmed death-1 (PD-1), T-cell immunoglobulin and mucin domain-containing protein-3 (TIM-3) and lymphocyte-activated gene-3 (LAG-3) (Rodrigues et al., 2014).

**Figure 5:**
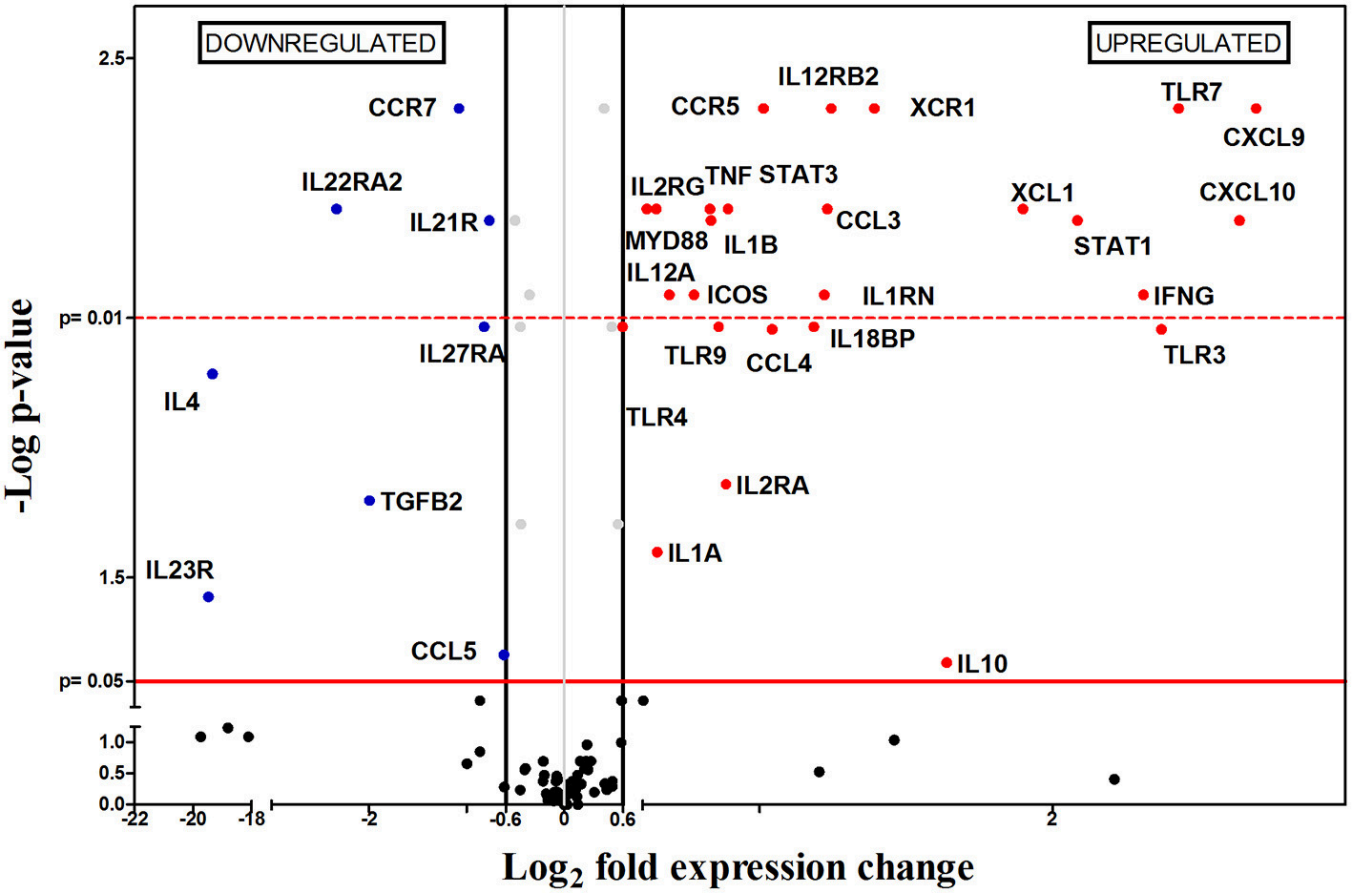
Differential gene-expression of the 112 analyzed genes in infected (*n* = 6) vs. control mice (*n* = 6), 8 wpi. The x-axis represents log_2_ of expression fold-change between infected and non-infected mice; the y-axis corresponds to the statistical significance, expressed as the negative logarithm of *p*-values. The red horizontal line indicates the cut-off for the statistical significance *p* = 0.05. Black vertical lines represent the log_2_ FC of −0.6 and 0.6 (corresponding to FC −1.5 and 1.5 respectively) used as biological threshold to identify differentially expressed genes. The negative values correspond to down-regulated genes (indicated in blue) and the positive values are the up-regulated genes (indicated in red). Black and gray dots represent non-differentially expressed genes.

Later in the chronic phase, 8 weeks after infection (8 wpi), 33 out of the 112 genes were differentially expressed between infected and non-infected mice. Unlike what happened 4 wpi, there was an overall upregulation in gene expression, with 25 upregulated and only 8 downregulated genes (Figure 5). The upregulated mRNAs encoded for chemokines (*Cxcl10, Cxcl9, Ccl3, Ccl4*, and *Xcl1*), chemokine receptors (*Ccr5* and *Xcr1*), interleukins (*Il1a, Il1b, Il10, Il12a, Il18bp*), interleukin receptors (*Il1rn, Il2ra, Il2rg, Il12rb2*), transcription factors (*Stat1, Stat3*), Toll-like receptors (*Tlr3, Ttlr4, Tlr7*, and *Tlr9*), cytokines (*Ifng, Tnfa*) and other genes (*Icos* and *Myd88*). Within the group of negatively regulated mRNAs, there were genes encoding for one chemokine (*Ccl5*), chemokine receptors (*Ccr7*), interleukins (*Il4*), four interleukin receptors (*Il21r, Il22ra2, Il23r*, and *Il27ra*) and one cytokine (*Tgfb2*). At the same time, clear differences in parasitic burden and weight are observed in infected spleens compared to healthy controls (Figures 1A,C).

Our analysis showed upregulation of 2 different chemokine receptors (*Ccr5* and *Xcr1)*, suggesting the recruitment of different cells including inflammatory monocytes (Gordon and Taylor, 2005; Gordon, 2007), Th1 lymphocytes, some DC (Bhattacharyya et al., 2008), NK cells (Liaskou et al., 2012), Treg cells (Yurchenko et al., 2006; Mougneau et al., 2011), and CD8^+^ and some DCs (Crozat et al., 2011) between 4 and 8 wpi; these cell populations are probably responsible for the splenomegaly observed at this point of the infection. Upregulation of *Ccr5* might be indicative of the presence of CCR5-expressing monocytes, which are highly susceptible to be infected by *Leishmania* (Bhattacharyya et al., 2008; Majumdar et al., 2014), and correlates with the 5000-fold increase of the parasite burden in the period between 4 and 8 wpi.

In addition to these chemokine receptors, other chemokine-encoding genes (*Ccl3, Ccl4, Cxcl9, Cxcl10*, and *Xcl1*) were induced by *L. infantum* infection at 8 wpi. CXCL9, and CXCL10 are chemoattractant for CXCR3-expressing cells (Kima and Soong, 2013), while both CCL3 and CCL4, are chemotactic for CCR5 expressing cells. The precise role of CCL3 remains unclear, but according to some authors it seems to be important in early containment of parasite burden and the generation of an anti-leishmanial cytokine environment, but may be deleterious in the latter stages of chronic *L. donovani* infection, since it promotes parasite persistence (reviewed in Oghumu et al., 2010). This effect can be seen in our experiment since, between weeks 4 and 8 post-infection, there was a significant increase of parasite burden. This cell recruitment produced an upregulation of *Leishmania*-induced proinflammatory genes (*Ifng, Tnfa, Il1a, Il1b*, and *Il18*) which also support the idea of an active inflammatory process at this point of the infection. Apart from these genes, there are other upregulated genes that are indirectly related to an inflammatory Th1-type response: *Tlr3, Tlr4*, and *Stat1* (Flandin et al., 2006; Schindler and Plumlee, 2008; Tuon et al., 2008; Singh et al., 2012).

However, even though there was an upregulation of inflammatory genes and *Ifng*, a Th1 characteristic gene, the immune system was unable to contain disease progression since parasite load keeps growing. One possible explanation for this can be found on the upregulation of *Il1rn* and *Il18bp*, coding for two anti-inflammatory cytokines which inhibit the proinflammatory effects produced by both IL-1a/IL-1b and IL-18, respectively (Correa and López, 2007). Another possibility is that IFN-γ is unable to exert its functions efficiently, for example by the existence of counteracting immune responses occurring at the same time. The lack of differences on mRNA expression levels of *Il5, Il13, Il17f, Il17a, Il27*, and *Foxp3*, along with downregulation of *Il4, Il23r, Il22ra2, Tgfbr2*, and *Tgfb2* preclude the generation of Th2, Th17, nTreg and iTreg responses. Similarly, upregulation of *FoxP3, Tgfb, Il0* and *Ctla4* has been associated to the development of CD4+CD25+ regulatory T cells (Tregs) (Yamashita et al., 2006), but those markers do not correlate in this study. Nevertheless, there is a non-FOXP3-expressing Treg subset known as Tr1 that may fit our expression profile by 4 reasons: (i) Tr1 cells co-express IFN-γ and IL-10 (Wakkach et al., 2003; Nylén et al., 2007; Gregori et al., 2012; Faleiro et al., 2014) and both genes are upregulated in our results; (ii) Tr1 differentiation is STAT3-mediated (Gregori et al., 2012) and this gene is upregulated; (iii) Tr1 cells do not produce IL-4 (Wu et al., 2007; Gregori et al., 2012), and *Il4* gene is downregulated by the *L. infantum*-infected group; (iv) *Icos* gene is upregulated and some authors indicate that ICOS is expressed in Tr1 cells (Häringer et al., 2009; Gregori et al., 2012). However, differentiation between a Tr1 population and other IL-10-producing T cell subsets is complicated, and other possibilities cannot be excluded (reviewed in Gregori et al., 2012).

An interesting possibility to explain the inability of the immune system to control disease progression is based on the upregulation of *Tnfa* and *Il10* and downregulation of *Ccr7*, and is consistent with previous works carried out by Stanley and coworkers (Stanley and Engwerda, 2006) using *L. donovani*-infected murine models. During chronic infection, the spleen suffers dramatic changes in microarchitecture, including disorganization of the white pulp, hypertrophy of the red pulp and disruption of the marginal zone (Kaye et al., 2004; reviewed in Rodrigues et al., 2016). These changes are related to a TNF-α-dependent, IL-10-mediated inhibition of CCR7 expression in DC, resulting in severely impaired DC migration to the periarteriolar lymphoid sheds (PALS) for antigen presentation to T cells, giving rise to a severe immunosuppression and enhancing parasite proliferation.

Both options, generation of Tr1 responses and alteration on the splenic architecture may be related events. In fact, *Il10* upregulation, as a compensatory mechanism to counteract an excess of TNF-α, might have its origin on regulatory DC (rDC) from the PALS. rDCs secrete IL-10 and skew T cell development to Tr1 cells, producing both IFN-γ and more IL-10 (Wakkach et al., 2003; Nylén et al., 2007; Gregori et al., 2012; Faleiro et al., 2014). IL-10 produced by both Tr1 and rDC cells, contributes to CCR7 downregulation hampering DC migration to the PALS, avoiding their contact with naïve T cells, and therefore blocking the establishment of antigen-specific T-cell responses.

### Mechanisms underlying leishmaniasis progression in spleen tissue over time

**Figure 6:**
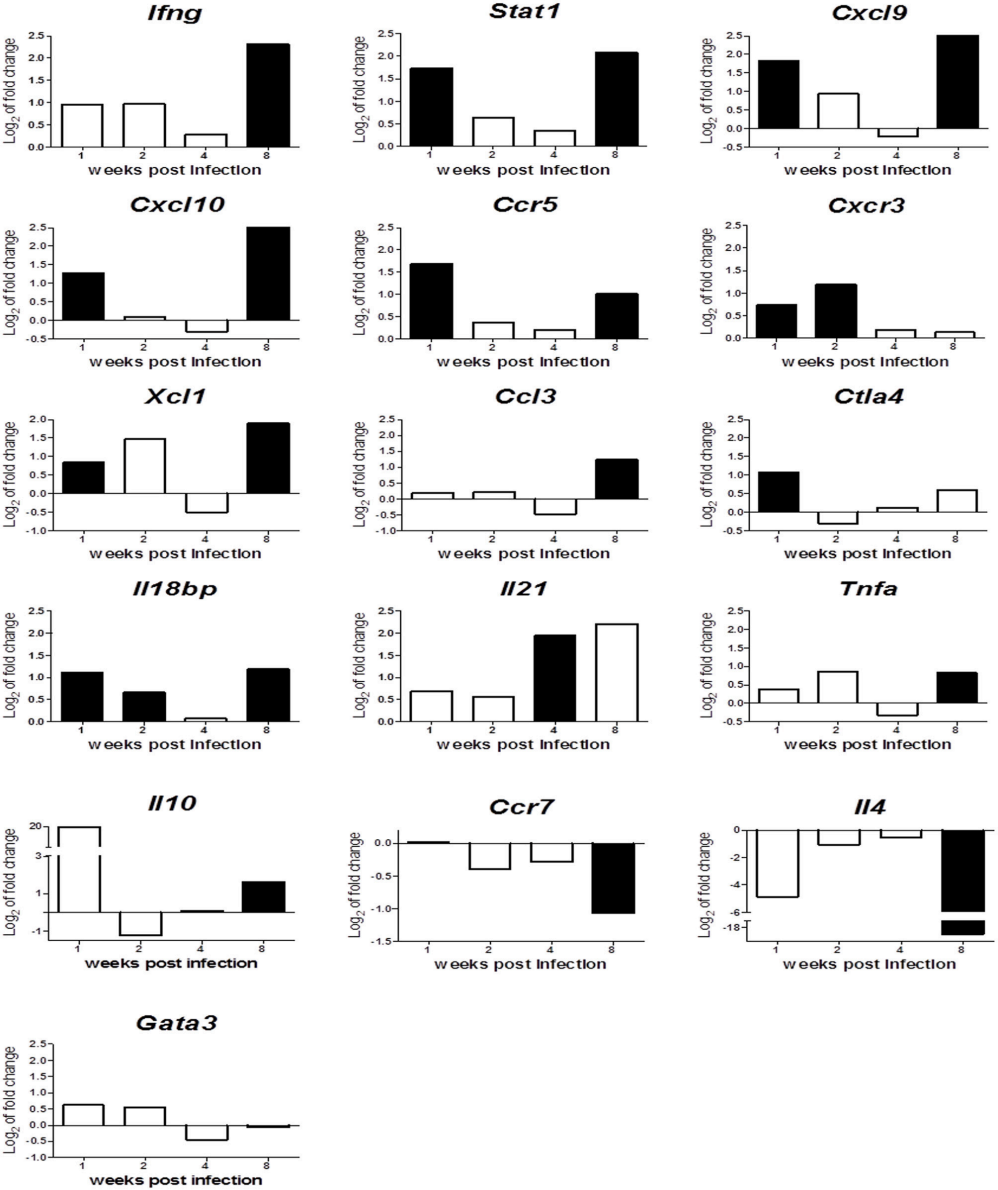
Relative gene expression of Th1, Th2 and immunoregulatory markers in spleens along infection. The y-axis represents log2 of expression fold-change for each indicated gene, that is the ratio between the average gene expression in the infected group and non-infected-control mice. The x-axis represents time after infection: 1, 2, 4, and 8 wpi. Solid black bars indicate statistically significant differences with *p* ≤ 0.05.

In order to identify the effect of *Leishmania* infection on expression of immune related genes over the course of infection, the expression fold-change between infected and control mice was plotted against time (Figure 6), indicating statistically significant differences (*p* < 0.05) between infected and control mice with black bars.

The analyses revealed that early response against *Leishmania* infection is characterized by the upregulation of Th1 markers and characteristic M1-macrophage activation molecules such as *Ifng, Stat1, Cxcl9, Cxcl10, Ccr5, Cxcr3, Xcl1*, and *Ccl3* (reviewed in Martinez and Gordon, 2014). This activation does not protect spleen from infection, since parasitic burden rises along time (Figure 1). This marked difference in gene expression between infected and control mice disappears during intermediate stages of infection (2 and 4 wpi). This inability to control infection and the loss of those Th1/M1 activation markers, may be related to strong anti-inflammatory and immunosuppresory signals that are activated early upon infection (*Ctla4*) or remain activated throughout the experiment (*Il18bp*). That would suggest that *L. infantum* might be blocking chemokine production to generate an adequate environment to maintain infection during these weeks, through a T-cell exhaustion process (Rodrigues et al., 2014), that the immune system tries to overcome with the strong upregulation of *Il21* at 4 wpi (Yi et al., 2010; Gigley et al., 2012; Wherry and Kurachi, 2015).

The overexpression of these Th1/M1 markers is restored later in the chronic phase (8 wpi), suggesting the generation of a classical “protective response” against leishmaniasis. Nonetheless, the parasitic burden rockets at this timepoint. This apparent contradiction can be explained by the generation of a Tr1 regulatory immune response characterized by overexpression of *Ifng, Tnfa, Il10* and downregulation *of Ccr7* and *Il4* (Figure 6), that counteracts the Th1/M1 response.

This global analysis of gene expression patterns during *Leishmania* infection in BALB/c spleen tissue raises two interesting points. Firstly, *Ifng* production is not a valuable predictor for Th1 protective responses, since its action may be counteracted in many different ways and might even take part in immunosuppresory mechanisms. Secondly, the classical Th2 response, characterized by IL-4 and its regulator GATA3 overexpression among other markers (reviewed in Selvapandiyan et al., 2012), is not playing any clear role in disease progression in this experimental model, given the downregulation of *Il4* and the lack of differential expression of *Gata3* gene observed.

Taken together, these results highlight the need for comprehensive analysis of gene expression in infected tissues or organs, in order to avoid misinterpretation of individual data. In practice, analyses of gene expression of a limited number of genes in complex scenarios like infection with an intracellular protozoan, might generate misleading results due to “missing information.”

This approach of using differential transcriptome analysis as a tool to understand Leishmania-host interactions has been successfully employed in several animal models and *in vitro* studies. Despite differences related to experimental models (Syrian hamster/mice/*in vitro* macrophages) and methodology (RNA-seq/qPCR/microarray hybridization) all of them draw a picture of mixed responses during infection and deactivation of effective parasite-controlling responses (Rabhi et al., 2012; Dillon et al., 2015; Fernandes et al., 2016; Kong et al., 2017; Medina-Colorado et al., 2017). Similar to our study, transcriptional profile of spleen samples from *L. donovani*-infected hamsters was analyzed 28 days after infection, revealing a strikingly proinflammatory environment and a strong expression of *Ifng* that did not protect against the increasing parasite burden (Kong et al., 2017). Likewise, chronic infection in hamsters revealed expression of markers of both T cell activation and inhibition, showing mixed expression of Th1 and Th2 cytokines and chemokines, and again ineffective in controlling infection (Medina-Colorado et al., 2017). Those studies that focused on the evaluation of early stages of infection (Dillon et al., 2015; Fernandes et al., 2016) revealed upregulation of both pro- and anti-inflammatory related genes similarly to our findings. In conclusion, the use of high-throughput technology on complex scenarios like the interaction between *Leishmania* and its animal host is opening new perspectives on immune response, and also providing large collections of data that can be useful for the identification of new potential biomarkers.

### Identification of potential biomarkers for leishmaniasis based upon linear and logistic regression models

Different approaches have been tested to identify new potential biomarkers able to predict infection, to determine parasitic load in infected organs or its clearance upon treatment. One interesting method is the use of multivariant statistical analyses to identify markers and to develop models able to predict disease parameters like infection (or absence of it) or parasitic burden.

The logistic regression model developed to determine whether there is a relationship among some of the genes whose expression has been analyzed in this work and absence/presence of parasite in the spleens, used normalized relative expression levels (NRQ) of the 36 genes coding for soluble markers (interleukins, cytokines…) from 47 randomly selected mice. The logistic regression model predicts the probability of infection in a given mouse, based on the expression levels (NRQ) of *Il18bp, Cxcl1* and *Il2*, being *Il18bp* and *Il2* directly correlated and *Cxcl1* inversely correlated (Table 1).

$$\begin{array}{l} p=\frac{1}{1+e^{-\left(-7.89+10.43*Il\text{18}p-5.35*Cxcl1+2.77*Il2\right)}} \end{array}$$

**Table 1:** Statistics of the logistic regression model.

**Variables (x)**	**β coefficients**	**Wald**	***p***
*Il18bp*	10.43	8.37	0.004
*Cxcl1*	−5.38	7.33	0.007
*Il2*	2.77	4.72	0.003
Constant	−7.89	7.5	0.006

*β coefficients for each selected variable, for the constant, and the results from Wald-tests including p-values*.

The predictive capacity of the model was auto-evaluated by comparing the observed results and those yielded by the model (Table 2).

**Table 2:** Logistic regression model auto-evaluation.

**Observed**		**Predicted**	
		**Infection**	
		**Absent**	**Present**
**Infection**	Absent	19 TN	4 FP
	Present	3 FN	21 TP

*TN, True Negatives. FP, False Positives. FN, False Negatives. TP, True Positives*.

The auto-evaluation of the proposed model classified correctly 40 out of the 47 samples (85%), with a sensitivity of 87.5% and a specificity of 82.6%. The effectiveness of the proposed model was evaluated using 14 extra mice that were not included on its development (Table 3). In this case, the sensitivity of the model was 57% and its specificity reached 85%.

**Table 3:** Logistic regression model evaluation.

**Observed**		**Predicted**	
		**Infection**	
		**Absent**	**Present**
**Infection**	Absent	6 TN	1 FP
	Present	3 FN	4 TP

*TN, True Negatives. FP, False Positives. FN, False Negatives. TP, True Positives*.

In the proposed model, the probability of infection has a positive correlation with the expression of genes coding for IL-18bp and IL-2 in spleen. IL-18bp is an inhibitor of the proinflammatory cytokine IL-18, which is a major inducing factor of IFN-γ, has multiple biological functions and is involved in immune regulation, anti-infection, and inflammation (Chaudhry et al., 2006). IL-18BP has been proposed as a biomarker of severity of injury after exposure to ionizing radiation in mice (Ha et al., 2016) and also a biomarker useful for differentiation of leptospirosis and dengue virus infection in humans (Conroy et al., 2014). IL-2 is and interleukin related with TL proliferation and the development of an adaptive immune response. IL-2 has been proposed as a valuable biomarker for detection of asymptomatic individuals in areas were *L. infantum* is endemic after whole blood stimulation with soluble *Leishmania* antigen (SLA), although the concentration of this biomarker is low (Ibarra-Meneses et al., 2016, 2017a). On the contrary, IL-2 did not perform as well for asymptomatic individuals from a *L. donovani* endemic area (Ibarra-Meneses et al., 2017b). In canine visceral leishmaniasis (CVL), serum IL-2 levels showed no correlation with disease severity (Solcà et al., 2016). Finally, in our model, expression of *Cxcl1* gene presents an inverse correlation with the probability of infection. CXCL1 chemokine plays a role in inflammation and is chemoattractant for neutrophils (reviewed in Kobayashi, 2008), the first-line defense against leishmania. Impaired CXCL1 levels have been related with increased susceptibility to *Klebsiella pneumoniae* in mice due to low inflammatory cell recruitment, reduced CXCL2 and CXCL5 production and decreased activation of NF-κB and MAPKs (Cai et al., 2010). Interestingly, CXCL1 serum levels in CVL were correlated with disease severity (Solcà et al., 2016).

Another interesting issue in leishmaniasis is the evaluation of parasitic load in infected animals, a useful parameter when evaluating treatment effectiveness. After analysis of the NRQ values of the 36 genes coding for soluble markers from 23 infected mice, a linear multivariant regression model was developed based on the expression levels of *Ccl3, Cxcl9*, and *Il18bp* (Table 4).

**Table 4:** Linear multivariant regression model.

	**β coefficient**	***T*-test**	***p*-value**	**Tolerance**	**Partial correlation**
*Ccl3*	1.971	6.367	0.00	0.615	−0.831
*Cxcl9*	−0.338	−5.052	0.00	0.749	−0.766
*Il18bp*	1.265	3.342	0.00	0.523	0.619
Constant	3.307	7.782	0.04		

The following equation predicts the parasitic burden in spleen from a given mouse based on the expression levels of *Ccl3, Cxcl9* and *Il18bp*, being *Il18bp* and *Ccl3* directly related and *Cxcl9* inversely correlated.

$$\begin{array}{l} \text{parasitic burden}=3.30+1.97*Ccl\text{3}-0.338*Cxcl\text{9}\\ +1.265*Il\text{18}bp \end{array}$$

ANOVA test showed *p* = 0.0, *R* = 0.94, and *R*^2^ = 0.89, indicating that the model is competent in the prediction of parasitic burden in spleen. The values of Tolerance and Partial correlation (Table 4) rejected co-linearity and partial correlation among the variables, therefore the three selected parameters are individually useful for parasitic burden determination. *Durbin Watson* test was 2.5, discarding independent errors.

It is interesting how the expression of these three genes may correlate with infection. *Ccl3* encodes CCL-3, a chemokine chemoattractant for macrophages, the preferred leishmania host-cells (Oghumu et al., 2010) and has been shown to be overexpressed in spleen during VL (Kong et al., 2017). CCL3 has been proposed as a biomarker for a series of conditions ranging from lymphoma (Takahashi et al., 2015) to osteoarthritis (Zhao et al., 2015) and chronic obstructive pulmonary disease (Ravi et al., 2014), in which inflammation plays a role. As indicated earlier, IL-18BP inhibits IL18-induced IFN-γ production in TL and the generation of an adaptive cellular response (Chaudhry et al., 2006). Therefore, it is likely that upregulation of both markers contributes to parasitic burden increase in spleen. The linear regression analysis selected *Cxcl9* gene expression as a marker negatively correlated with parasitic burden in spleen. Given its role on immunopathogenesis of the disease in mice, recruiting lymphocytes toward spleen, the selection of this marker is not surprising, and its overexpression has been reported in spleen during VL (Kong et al., 2017).

The competence of the proposed model was evaluated using data from 8 extra mice that were not included in its development, as described in the Methods section. As shown in Table 5, the fitting of the model is good, since the difference between estimated and observed parasitic loads is low (<1.5 log units) in 6 out of 8 animals and the observed burden was inside the confidence interval in all of them.

**Table 5:** Observed and estimated parasitic burden (expressed as Log_10_) in the infected spleens.

**Sample mouse**	**Observed load (Log)**	**Estimated load (Log)**	**Upper limit (Log)**	**Lower limit (Log)**
1	7.27	5.81	7.90	3.71
2	5.55	5.65	7.73	3.58
3	6.74	6.63	9.09	4.18
4	7.25	6.25	8.52	3.99
5	7.28	6.14	8.55	3.73
6	8.15	10.17	15.74	4.59
7	7.73	5.64	7.88	3.40
8	12.96	13.67	20.02	7.38

*Upper and lower limits indicate 95% confidence intervals of the estimated parasitic burden*.

The gene expression of the majority of the variables selected in these regression models as relevant for disease detection or progression have not been studied in pathological situations like *Leishmania* infection. Gene expression does not always translate into protein expression, and soluble factors, such as CCL3, IL-18BP or CXCL9, may or may not be secreted into blood or plasma in a fashion that correlates with gene expression. Nevertheless, the levels in serum, plasma or stimulated blood cells of some of them have been proposed as biomarkers during different conditions, suggesting that the use of some of these molecules may be useful for the monitorization of different aspects of leishmania infections in experimental models. More research will be needed to assess the real practical value of the biomarkers and the prediction models described in this manuscript, such as the actual expression levels of the proteins or their specificity during infection, but our findings outline an innovative strategy for identification of new potential biomarkers in visceral leishmaniasis.

## Data availability statement

The data discussed in this publication have been deposited in NCBI's Gene Expression Omnibus (Edgar, 2002) and are accessible through GEO Series accession number GSE112129 (for the infection experiments) (https://www.ncbi.nlm.nih.gov/geo/query/acc.cgi?acc=GSE112129) and GSE112138 (for the identification of potential biomarkers) (https://www.ncbi.nlm.nih.gov/geo/query/acc.cgi?acc=GSE112138).

## Author contributions

EO and YH-S performed the experiments and the statistical analysis. EC, AG-G and BV contributed to conception and design of the study. AG-G and ML contributed to establishing the experimental infection model. EO wrote the first draft of the manuscript. EC wrote the final version of the manuscript. BV, ML contributed to discussion and analysis of data. All authors contributed to manuscript revision, read and approved the submitted version.

### Conflict of interest statement

The authors declare that the research was conducted in the absence of any commercial or financial relationships that could be construed as a potential conflict of interest.

## Footnotes

^1^Crt, a relative threshold method for qPCR data analysis on the QuantStudioTM 12K Flex system with OpenArray® technology (2014). Appl. Biosyst. QuantStudioTM 12K Flex Real-Time PCR Syst. Appl. Note CO28730, 4.
