# Supplementary material for: Transcriptional Profiling of Immune-Related Genes in Leishmania infantum-Infected Mice: Identification of Potential Biomarkers of Infection and Progression of Disease
Source: Front Cell Infect Microbiol. 2018 Jun 26;8:197. doi: 10.3389/fcimb.2018.00197 (PMC6036295; doi:10.3389/fcimb.2018.00197)
Supplement: Supplementary file 1 [file Table_1.PDF]

Table S1. List of 112 TaqMan assays used for RT-qPCR analysis using QuantStudio™ 12K Flex Real-Time PCR System. Genes coding for soluble molecules (n=36) used in the regression models are indicated in Italics.

Nº	Gene Symbol	Assay ID	Gene Name
1	*Arg1*	*Mm00475988_m1*	*Arginase, liver*
2	B2m	Mm00437762_m1	Beta-2 microglobulin
3	*Ccl17*	*Mm00516136_m1*	*Chemokine (C-C motif) ligand 17*
4	Ccl2	Mm00441242_m1	Chemokine (C-C motif) ligand 2
5	Ccl22	Mm00436439_m1	Chemokine (C-C motif) ligand 22
6	*Ccl3*	*Mm00441258_m1*	*Chemokine (C-C motif) ligand 3*
7	*Ccl4*	*Mm00443111_m1*	*Chemokine (C-C motif) ligand 4*
8	*Ccl5*	*Mm01302427_m1*	*Chemokine (C-C motif) ligand 5*
9	*Ccl7*	*Mm00443113_m1*	*Chemokine (C-C motif) ligand 7*
10	Ccr1	Mm01216147_m1	Chemokine (C-C motif) receptor 1
11	Ccr2	Mm01216173_m1	Chemokine (C-C motif) receptor 2
12	Ccr4	Mm00438271_m1	Chemokine (C-C motif) receptor 4
13	Ccr5	Mm01216171_m1	Chemokine (C-C motif) receptor 5
14	Ccr7	Mm01301785_m1	Chemokine (C-C motif) receptor 7
15	Cd28	Mm00483137_m1	CD28 antigen
16	Cd40	Mm00441891_m1	CD40 antigen
17	Cd40lg	Mm00441911_m1	CD40 ligand
18	Cd54	Mm00516023_m1	Intercellular adhesion molecule 1
19	Cd80	Mm00711660_m1	CD80 antigen
20	Cd86	Mm00444543_m1	CD86 antigen
21	Ctla4	Mm00486849_m1	Cytotoxic T-lymphocyte-associated protein 4
22	*Cxcl1*	*Mm04207460_m1*	*Chemokine (C-X-C motif) ligand 1*
23	*Cxcl10*	*Mm00445235_m1*	*Chemokine (C-X-C motif) ligand 10*
24	*Cxcl2*	*Mm00436450_m1*	*Chemokine (C-X-C motif) ligand 2*
25	*Cxcl9*	*Mm00434946_m1*	*Chemokine (C-X-C motif) ligand 9*
26	Cxcr2	Mm00438258_m1	Chemokine (C-X-C motif) receptor 2
27	Cxcr3	Mm00438259_m1	Chemokine (C-X-C motif) receptor 3
28	Cxcr4	Mm99999055_m1	Chemokine (C-X-C motif) receptor 4
29	*Ebi3*	*Mm00469294_m1*	*Epstein-Barr virus induced gene 3*
30	Foxp3	Mm00475162_m1	Forkhead box P3
31	Gata3	Mm00484683_m1	GATA binding protein 3
32	Hprt	Mm00446968_m1	Hypoxanthine guanine phosphoribosyl transferase
33	Icam2	Mm00494862_m1	Iintercellular adhesion molecule 2
34	Icos	Mm00497600_m1	Inducible T cell co-stimulator
35	Icosl	Mm00497237_m1	Icos ligand
36	*Ifng*	*Mm01168134_m1*	*Interferon gamma*
37	Ifngr1	Mm00599890_m1	Interferon gamma receptor 1
38	Ifngr2	Mm00492626_m1	Interferon gamma receptor 2
39	*Il10*	*Mm00439614_m1*	*Interleukin 10*
40	Il10ra	Mm00434151_m1	Interleukin 10 receptor, alpha
41	Il10rb	Mm00434157_m1	Interleukin 10 receptor, beta
42	*Il12a*	*Mm00434165_m1*	*Interleukin 12a*
43	*Il12b*	*Mm00434174_m1*	*Interleukin 12b*
44	Il12rb1	Mm00434189_m1	Interleukin 12 receptor, beta 1

45	Il12rb2	Mm00434200_m1	Interleukin 12 receptor, beta 2
46	Il13	Mm00434204_m1	Interleukin 13
47	Il13ra1	Mm00446726_m1	Interleukin 13 receptor, alpha 1
48	Il13ra2	Mm00515166_m1	Interleukin 13 receptor, alpha 2
49	Il17a	Mm00439618_m1	Interleukin 17A
50	*Il17f*	*Mm00521423_m1*	*Interleukin 17F*
51	Il17ra	Mm00434214_m1	Interleukin 17 receptor A
52	*Il18*	*Mm00434225_m1*	*Interleukin 18*
53	*Il18bp*	*Mm00456733_m1*	*Interleukin 18 binding protein*
54	Il18r1	Mm00515178_m1	Interleukin 18 receptor 1
55	Il18rap	Mm00516053_m1	Interleukin 18 receptor accessory protein
56	*Il1a*	*Mm00439620_m1*	*Interleukin 1 alpha*
57	*Il1b*	*Mm00434228_m1*	*Interleukin 1 beta*
58	Il1r1	Mm00434237_m1	Interleukin 1 receptor, type I
59	Il1rap	Mm00492638_m1	Interleukin 1 receptor accessory protein
60	Il1rn	Mm00446186_m1	Interleukin 1 receptor antagonist
61	*Il2*	*Mm00434256_m1*	*Interleukin 2*
62	*Il21*	*Mm00517640_m1*	*Interleukin 21*
63	Il21r	Mm00600319_m1	Interleukin 21 receptor
64	Il22	Mm00444241_m1	Interleukin 22
65	Il22ra1	Mm01192943_m1	Interleukin 22 receptor, alpha 1
66	Il22ra2	Mm01192969_m1	Interleukin 22 receptor, alpha 2
67	Il23a	Mm00518984_m1	Interleukin 23, alpha subunit p19
68	Il23r	Mm00519943_m1	Interleukin 23 receptor
69	*Il27*	*Mm00461162_m1*	*Interleukin 27*
70	Il27ra	Mm00497259_m1	Interleukin 27 receptor, alpha
71	Il2ra	Mm01340213_m1	Interleukin 2 receptor, alpha chain
72	Il2rb	Mm00434268_m1	Interleukin 2 receptor, beta chain
73	Il2rg	Mm00442885_m1	Interleukin 2 receptor, gamma chain
74	*Il4*	*Mm00445259_m1*	*Interleukin 4*
75	Il4ra	Mm01275139_m1	Interleukin 4 receptor, alpha
76	*Il5*	*Mm00439646_m1*	*Interleukin 5*
77	Il5ra	Mm00434284_m1	Interleukin 5 receptor, alpha
78	*Il6*	*Mm00446190_m1*	*Interleukin 6*
79	Il6ra	Mm00439653_m1	Interleukin 6 receptor, alpha
80	Il6st	Mm00439665_m1	Interleukin 6 signal transducer
81	Itgal	Mm00801807_m1	Integrin alpha L
82	Itgam	Mm00434455_m1	Integrin alpha M
83	Itgb2	Mm00434513_m1	Integrin beta 2
84	Myd88	Mm00440338_m1	Myeloid differentiation primary response gene 88
85	*Nos2*	*Mm00440502_m1*	*Nitric oxide synthase 2, inducible*
86	*Nox4*	*Mm00479246_m1*	*NADPH oxidase 4*
87	Pgk1	Mm00435617_m1	Phosphoglycerate kinase 1
88	Polr2a	Mm00839493_m1	Polymerase (RNA) II (DNA directed) polypeptide A
89	*Ptges*	*Mm00452105_m1*	*Prostaglandin E synthase*
90	*Ptgs2*	*Mm00478374_m1*	*Prostaglandin-endoperoxide synthase 2*
91	Stat1	Mm00439531_m1	Signal transducer and activator of transcription 1
92	Stat3	Mm01219775_m1	Signal transducer and activator of transcription 3

93	Stat4	Mm00448890_m1	Signal transducer and activator of transcription 4
94	Stat5a	Mm00839861_m1	Signal transducer and activator of transcription 5A
95	Stat5b	Mm00839889_m1	Signal transducer and activator of transcription 5B
96	Stat6	Mm01160477_m1	Signal transducer and activator of transcription 6
97	Tbp	Mm00446971_m1	TATA box binding protein
98	*Tgfb1*	*Mm01178820_m1*	*Transforming growth factor, beta 1*
99	*Tgfb2*	*Mm00436955_m1*	*Transforming growth factor, beta 2*
100	Tgfbr1	Mm00436964_m1	Transforming growth factor, beta receptor I
101	Tgfbr2	Mm00436977_m1	Transforming growth factor, beta receptor II
102	Tlr2	Mm00442346_m1	Toll-like receptor 2
103	Tlr3	Mm01207404_m1	Toll-like receptor 3
104	Tlr4	Mm00445273_m1	Toll-like receptor 4
105	Tlr7	Mm00446590_m1	Toll-like receptor 7
106	Tlr9	Mm00446193_m1	Toll-like receptor 9
107	*Tnf*	*Mm00443258_m1*	*Tumor necrosis factor*
108	Tnfrsf1a	Mm00441875_m1	Tumor necrosis factor receptor superfamily, member 1a
109	Tnfrsf1b	Mm00441889_m1	Tumor necrosis factor receptor superfamily, member 1b
110	Ubc	Mm01201237_m1	Ubiquitin C
111	*Xcl1*	*Mm00434772_m1*	*Chemokine (C motif) ligand 1*
112	Xcr1	Mm00442206_s1	Chemokine (C motif) receptor 1
